# Transcutaneous Auricular Vagus Nerve Stimulation Improves Inflammation but Does Not Interfere with Cardiac Modulation and Clinical Symptoms of Individuals with COVID-19: A Randomized Clinical Trial

**DOI:** 10.3390/life12101644

**Published:** 2022-10-19

**Authors:** Fernanda Ishida Corrêa, Paulo Henrique Leite Souza, Laura Uehara, Raphael Mendes Ritti-Dias, Gustavo Oliveira da Silva, Wellington Segheto, Kevin Pacheco-Barrios, Felipe Fregni, João Carlos Ferrari Corrêa

**Affiliations:** 1 Doctoral and Master’s Programs in Rehabilitation Sciences, Nove de Julho University, São Paulo 01525-000, Brazil; 2 Neuromodulation Center and Center for Clinical Research Learning, Spaulding Rehabilitation Hospital and Massachusetts General Hospital, Harvard Medical School, Boston, MA 02114, USA; 3 Unidad de Investigación para la Generación y Síntesede Evidencias en Salud, Universidad San Ignacio de Loyola, Lima 15024, Peru

**Keywords:** COVID-19, vagus nerve stimulation, inflammation, autonomic modulation, clinical symptoms

## Abstract

Transcranial auricular vagus nerve stimulation (taVNS) has shown effectiveness in reducing inflammation and depression. Thus, this study evaluated its effect on inflammation, cardiac autonomic modulation, and clinical symptoms in individuals affected by COVID-19. **Methods**: There were 52 randomized participants hospitalized with COVID-19 diagnosis who were to receive active (a-taVNS) or sham taVNS (s-taVNS) for 90 min twice a day for seven consecutive days. Interleukin 6 (IL-6), 10 (IL-10), cortisol, C-reactive protein (CRP), heart rate variability (HRV), and clinical symptoms were assessed before and after seven days of treatment. There were also seven- and fourteen-day follow-ups for clinical symptoms, including anxiety and depression levels, as well as a six-month follow-up for memory and attention levels. **Results**: There was significant reduction in CRP −23.9%, (95% CI −46.3 to −1.4) and IL-6 −37.7%, (95% CI −57.6 to −17.7) for the a-taVNS group. There were no changes in IL-10, cortisol levels, or in HRV results (*p* > 0.05) in both groups. There were no changes regarding clinical symptoms, except for a significant decrease in depression level (−2.85, 95% CI −5.44 to −0.27) in the a-taVNS group. **Conclusion**: taVNS showed effects on CRP, IL-6, and depression levels; however, it did not affect other clinical symptoms.

## 1. Introduction

The clinical features of COVID-19 vary from an asymptomatic state to a severe clinical status such as pneumonia and death [1]. COVID-19 is associated with an extreme increase in inflammatory cytokines in the blood, known as a cytokine storm [2]. It has also been observed that people with COVID-19 have sympathetic hyperactivity and impaired parasympathetic autonomic signaling that can affect the cardiovascular system [3].

Thus, treatment to minimize inflammation and its clinical effects has been implemented for patients, using corticosteroids and ventilatory support [4,5]. However, complementary therapies have been investigated to potentiate the effects of these treatments, such as transcutaneous auricular vagus nerve stimulation (taVNS).

taVNS is a powerful tool for modulating bodily functions, as the vagus nerve is an important neuro immunomodulator in inflammatory body processes [6,7]. The use of taVNS for patients diagnosed with COVID-19 was recently researched [8,9], showing its effectiveness for reducing inflammatory marker levels and managing symptoms and depression. This non-invasive stimulation may also exhibit favorable cardiovascular effects during sepsis [10].

Thus, this study aimed to evaluate the effect of taVNS on inflammation, cardiac autonomic modulation and clinical symptoms, including levels of anxiety, depression, attention, and memory of patients diagnosed with Covid-19.

## 2. Material and Methods

Participants were randomized (www.randomizer.org) by a researcher not involved in the treatments for 14 sessions of active transcutaneous auricular vagus nerve stimulation (a-taVNS) or sham transcutaneous auricular vagus nerve stimulation (s-taVNS) (Appendix A). The participants were blind to the treatment.

The local Ethics Committee for Research on Human Subjects approved this study (CAAE: 46699521.5.0000.5511) in compliance with Resolution 466/12 of the Brazilian National Board of Health. All participants signed an informed consent document before the beginning of the study. The protocol was registered in the Brazilian Registry of Clinical Trials (RBR–399t4g5).

### 2.1. Participants

Physiotherapists selected as participants adults admitted to Lydia Storópoli Hospital who had been diagnosed with moderate or severe COVID-19. To be included, participants had to have symptoms of COVID-19 within ten days of the beginning of the first evaluation of this research. Individuals who had contraindications for the use of taVNS (cochlear implants and cardiac pacemakers) or who were not conscious enough to consent to taVNS treatment and to respond to the initial questionnaires were not included.

**Figure 1:**
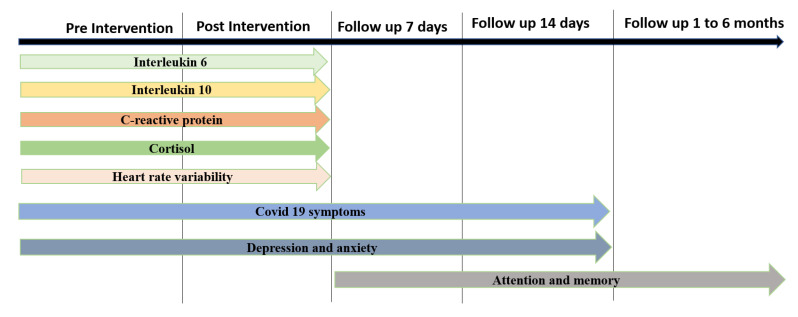
Timeline of Outcome Measures. Source: authors.

The recruited participants were evaluated for their inflammatory profile, cardiac autonomic modulation, and clinical symptoms before and after the interventions. Seven and fourteen days after the end of the interventions, clinical symptoms (including depression, anxiety, attention, and memory) were reassessed. The level of attention and memory was further monitored during a 6-month follow-up (Figure 1).

### 2.2. Intervention

#### 2.2.1. Experimental Group

Participants continued to receive usual medical care throughout the study, as prescribed by the hospital physician. In addition, participants in the experimental group received transcutaneous auricular vagus nerve stimulation (taVNS) twice a day for seven consecutive days, totaling 14 stimulation sessions, with 90 min of stimulation for each session.

The a-taVNS was applied using a multifunctional transcutaneous neuromuscular electrical stimulator (model Dualpex 071, Quark Produtos Médicos), with a current ranging from 25 Hz to 5 kHz, by series of sine waves, with a pulse of 1 ms. Intensity was adjusted based on the participant’s tolerance in order to not cause pain or muscle contractions. One 15 mm electrode was positioned on the tragus [11] from the left ear and another on the left clavicle.

#### 2.2.2. Control Group

Participants continued receiving their usual medical care throughout the study, as prescribed by the hospital physician. For sham stimulation, the same protocol as the a-taVNS was used; however, the equipment remained off.

### 2.3. Outcome Measures

#### 2.3.1. Primary Outcome Measures—Inflammatory Profile

To obtain the inflammatory profile, 15 mL of blood was taken via vein puncture, always in the morning. It was stored in a tube containing protease inhibitor and EDTA, then centrifuged at 3000 rpm for 15 min at 4 °C. The resulting plasma was transferred to micro centrifuge tubes (1.5 mL) and stored at −80 °C. This sample was later sent for analysis of Interleukin-6 (IL-6), Interleukin-10 (IL-10), Cortisol, and C-reactive Protein (CRP).

IL-6, IL-10, and cortisol were measured by the Enzyme-Linked Immuno-Sobent Assay (ELISA) method and Chemiluminescent Competitive Immunoassay following the manufacturer’s recommendations. The quantitative determination of CRP was performed by nephelometry (Dade-Behring N High Sensitivity CRP).

#### 2.3.2. Secondary Outcomes Measures

##### Cardiac Autonomic Modulation

Cardiac autonomic modulation was analyzed using heart rate variability (HRV), with a heart rate monitor (V800, Polar Electro, Finland). RR intervals were recorded for 15 min, and those with at least five minutes of the stationary signal were considered valid.

The RR intervals were exported to the Kubios HRV program (Version 2.0, Biosignal Analysis and Medical Imaging Group, Finland) for analysis of the frequency domain (low-frequency–LF, high-frequency–HF, and low-frequency and high-frequency ratio–LF/HF).

Frequency domain parameters were obtained through spectral analysis using the autoregressive method. Analysis of all parameters followed the standards of the Task Force of the European Society of Cardiology and the North American Society of Pacing and Electrophysiology [12] and updated recommendations [13].

##### COVID-19 Clinical Symptoms

To obtain the frequency and intensity of COVID-19 clinical symptoms a questionnaire developed by the authors was applied. The list of symptoms in the questionnaire was based on Yang et al. [14] and Umakanthan et al. [15]. Possible responses were none, mild, moderate, or severe.

Personal information and health condition were collected from participants’ medical records. After the end of treatment, if the participants were discharged from the hospital, they were contacted by telephone.

##### Depression and Anxiety Scale

These variables were assessed by the Hospital Anxiety and Depression Scale [16]; results were classified as unlikely (score 0–7), possible–questionable or doubtful (8–11 points), and probable (12–21 points).

##### Attention and Memory Levels

A questionnaire adapted from the Clinical Global Impression Scale (CGI) [17] was applied to obtain these data. Possible answers were much better, better, a little better, no change, a little worse, worse, and much worse.

### 2.4. Data Analysis

The sample size calculation was performed using GPOWER3 Software, with 21 subjects (11 from the s-taVNS and 10 from the a-taVNS). The results of pre-and post-intervention differences of primary (IL-6) and secondary (LF/HF) outcomes were considered for analysis. Considering a power of 80%, an α error of 5%, and an effect size of 1.0403050, the estimated sample size was 26 per group for primary outcomes; for secondary outcomes the estimated sample size was 14 patients per group, considering an effect size of 0.9735431.

Primary outcomes were the difference in CRP, IL-6, IL-10, and cortisol from baseline to the last day of stimulation. Clinical outcomes (anxiety, depression, CGI memory, CGI attention, HRV, duration of hospitalization, and mortality) were considered secondary outcomes.

Baseline characteristics were reported with descriptive statistics for each group. Two-tailed tests were applied in all analyses. A significance level of 0.05 and an intention-to-treat (ITT) analysis were used. To manage missing data, the last observation performed method was employed. The assumption of normality was tested using histograms and the Shapiro–Wilk test.

First, changes in inflammatory mediators were standardized by baseline values using the following formula:

$$\%\;\mathrm{of}\;\mathrm{chance}\;\mathrm{of}\;\mathrm{inflammatory}\;\mathrm{marker}=\frac{\left(\mathrm{post}\;\mathrm{treatment}\;\mathrm{value}-\mathrm{Baseline}\right)}{\mathrm{post}\;\mathrm{treatment}\;\mathrm{value}\;}\times 100$$

Then, generalized linear models with identity functions were implemented to test the change in outcomes across groups (post-treatment–baseline). Unbalanced factors were identified at baseline (vaccination status, anxiety score, sex, and body mass index). These variables were, therefore, included as covariates for adjustment.

Durbin–Watson estimates and Cook’s distance values were used for regression diagnostics analysis, such as residual autocorrelation and influential cases. The normality of residuals was assessed using QQ-plots. Homoscedasticity assumptions were visually verified by plotting residuals against predicted values. No correction for multiple comparisons was performed to decrease the risk of type II errors in this exploratory analysis, although the number of multiple comparisons was minimized following a predefined list of outcomes. All analyses were performed in R version 4.2.0. The HF (n.u.) and LF (n.u.) and HF/LF ratio analyzed HRV; analysis was performed using SPSS version 22.0 for Windows. Generalized Estimating Equations analyzed HRV, memory CGI, and attention CGI data. A value of *p* < 0.05 was considered significant.

## 3. Results

A total of 84 participants admitted to Lydia Storópoli Hospital from May 2021 to December 2021 were screened; 32 participants were excluded, seven for not meeting the eligibility criteria, seven who died during treatment, 16 due to hospital discharge during taVNS treatment, and two due to intubation before the first assessments and randomization. Thus, 52 participants remained for analysis. The participants’ demographic data are shown in Table 1.

The a-taVNS group had more male participants than the s-taVNS group; a higher level of anxiety was observed for a-taVNS group participants. Most participants in both groups were overweight. The s-taVNS group had more participants with chronic obstructive pulmonary disease and more who were vaccinated. The groups were equal at baseline to HRV.

They were continuously medicated to reduce the symptoms of COVID-19 during this research and, thus, did not just receive the taVNS treatment. In Appendix B there is information about the medical treatment in use during the trial for both groups. There is no significant difference between them.

### 3.1. Effects of Intervention

#### 3.1.1. Primary Outcome–Inflammatory Profile

**Figure 2:**
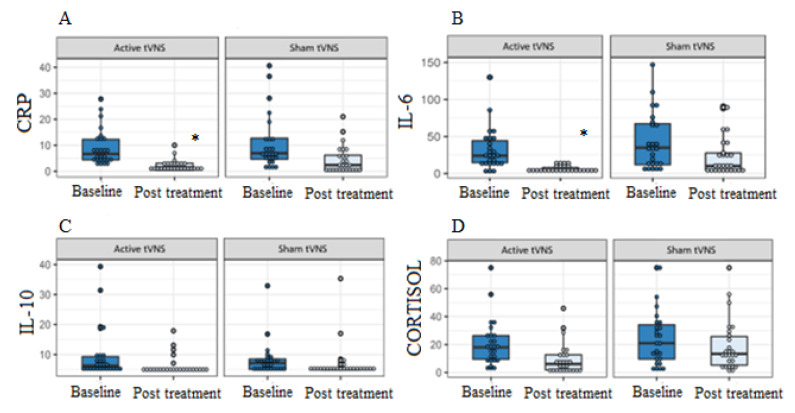
Effects of transcutaneous auricular vagus nerve stimulation (taVNS) on CRP (C-reactive protein) levels (**A**), IL-6 (interleukin 6) (**B**), IL-10 (interleukin 10) (**C**), cortisol (**D**) at baseline and after 14 sessions of taVNS, for groups a-taVNS (*n* = 26) and s-taVNS (*n* = 26). * *p* < 0.05.

There was a significant reduction in CRP (difference between groups = −23.9%, 95% CI −46.3 to −1.4, *p* = 0.038) and IL-6 (difference between groups = −37.7%, 95% CI −57.6 to −17.7, *p* < 0.001) percentage post treatment with a-taVNS, after adjusting for unbalanced variables at baseline (vaccination status, anxiety score, sex, and body mass index) (Figure 2).

#### 3.1.2. Secondary Outcomes

##### Heart Rate Variability (HRV)

**Figure 3:**
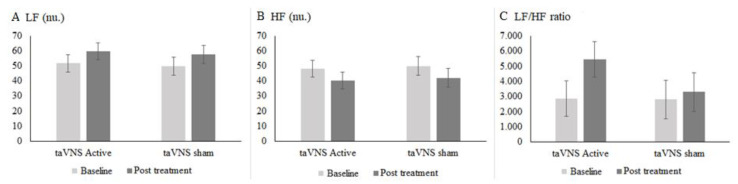
Effects of taVNS at baseline and after 14 sessions of taVNS on the variables LF (Low Frequency) (**A**); HF (High Frequency) (**B**); HF/LF (High Frequency and Low Frequency Ratio) (**C**) for the a-taVNS (*n* = 24) and s-taVNS (*n* = 21) groups. n.u (Standardized Units). Data Expressed as mean and standard deviation (SD), the Generalized Estimating Equations (GEE) test was used.

Two participants from the a-taVNS and five participants from the s-taVNS were excluded, as the HR signals were inadequate for analysis; thus, 24 a-taVNS participants and 21 s-taVNS were considered (Figure 3).

Figure 3 shows the results of the LF band (n.u), (difference intergroup: 95% IC 40.5 to 62.9) for a-taVNS in pre-treatment vs. post-treatment (95% IC 48.4 to 70.1) and in the s-taVNS, values were (95% IC 37.7 to 61.6) pre-treatment vs. post-treatment (95% IC 45.7 to 69.6). There was no significant difference between groups in post-treatment (95% IC −14.4 to 18.4, *p* = 0.812).

The pre-treatment HF values (n.u) for the a-taVNS were (95% IC 36.9 to 59.2) vs. post-treatment (95% IC 29.1 to 51.4), and in the s-taVNS, pre-treatment HF was (95% IC 38.0 to 61.9) vs. post-treatment (95% IC 30.1 to 54.0), with no significant difference between the groups in post-treatment (95% IC −18.1 to 14.5, *p*= 0.829).

The LF/HF values for a-taVNS pre-treatment were (95% IC 566.4 to 5177.6) vs. post-treatment (95% IC 3150.4 to 7759.6), and for s-taVNS pre-treatment LF/HF was (95% IC 332.5 to 5260) vs. post-treatment (95% IC 825.9 to 5753.3), without a significant difference between the groups (95% IC −1208.2 to 5538.0 *p* = 0.208).

##### Clinical Symptoms

**Figure 4:**
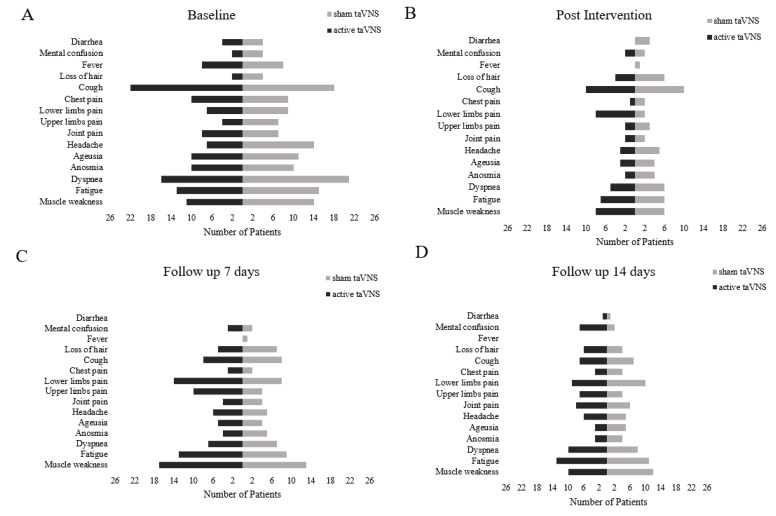
Clinical symptoms of the a-taVNS (N = 26) and s-taVNS (N = 26) groups pre-treatment (**A**); post-treatment (**B**); 7 days of follow-up (**C**); 14 days of follow-up (**D**). Transcutaneous auricular vagus nerve stimulation *(taVNS)*.

The most frequent initial clinical symptoms for the a-taVNS group were cough, dyspnea, fatigue, and muscle weakness. For the s-taVNS group, those symptoms were cough, dyspnea, migraine, ageusia, fatigue, and muscle weakness. After treatment, there was a decrease in clinical symptoms in both groups, but compared to baseline, the a-taVNS group had more participants with clinical symptoms than the s-taVNS (Figure 4).

Of the 26 participants who started treatment, only 20 participants per group responded to the questionnaire at follow-up, as the others progressed to intubation.

At the 7-day follow-up, both groups experienced an increase in clinical symptoms. The most frequent clinical symptoms in the a-taVNS group were lower and upper limb pain, fatigue, and muscle weakness; s-taVNS presented cough, pain in lower limbs, fatigue, and muscle weakness. Nineteen participants in the a-taVNS group and 20 in the s-taVNS completed the assessment, as five participants in the a-taVNS group and four in the s-taVNS group remained unconscious, and two participants in each group died.

In the 14-day follow-up, there was a decrease in clinical symptoms, with mental confusion and fatigue being more frequent in the a-taVNS group, and lower limb pain, fatigue, and muscle weakness in the s-taVNS group. In both groups, there was an increase in diarrhea symptoms regarding the seventh day; 20 per group completed the clinical assessment, four participants in the a-taVNS group and two participants in the s-taVNS group remained unconscious, and six participants died (two from a-taVNS and four from s-taVNS).

##### Depression and Anxiety

There was a statistically significant reduction in depression in a-taVNS compared to s-taVNS (difference between groups = −2.85, 95% CI −5.44 to −0.27, *p* = 0.03), but no changes in anxiety levels (Table 2).

##### Attention and Memory Level

Twenty participants from each group were analyzed because five participants died in the a-taVNS group and one was not contacted for follow-up. In the s-taVNS group, six participants died during follow-up (Table 3).

There was no statistically significant difference in the attention and memory levels in the follow-up for both groups. During the six-month follow-up, four participants from the a-taVNS group and five from s-taVNS group reported improvement in memory in the fourth month after diagnosis of COVID-19; eight participants from the a-taVNS group and six from s-taVNS group reported memory getting worse after the fourth month.

Regarding attention, eight participants in the a-taVNS group and seven in the s-taVNS group reported improvement after the fourth month following COVID-19 diagnosis, and eight participants in the a-taVNS group and five in the s-taVNS group reported a worsening from the fourth month after COVID-19 diagnosis.

## 4. Discussion

### 4.1. Inflammatory Mediators

Our results showed a decrease in CRP and IL-6 levels after taVNS. However, no difference was found in IL-10 and cortisol levels. Tornero et al. [8] observed significant reductions in CRP in participants with COVID-19 who received medical treatment associated with nVNS. However, their participants received two consecutive two-minute sessions of nVNS, three times a day in one day of treatment, whereas our participants received 90 min of stimulation, two times a day during 7 days. Another study [9] observed a decrease in IL-6 in two patients with COVID-19 after receiving taVNS, with, however, 60 min of stimulation per day, for around 17 days.

Almost all participants in our study had CRP greater than 1.0 mg/d pre-treatment; after treatment, there was a significant decrease in CRP levels, but some participants in both groups still had CRP levels above 1.00 mg/dL post-treatment, which indicates inflammatory processes [18].

The anti-inflammatory effect of VNS has been documented for different diseases, such as Crohn’s disease [19], Rheumatoid Arthritis [20], and Sjögren’s syndrome [21]. Therefore, using VNS for inflammation treatment appears to be feasible, especially in COVID-19, the main characteristic of which is the inflammatory process. This theoretical concept of the VNS anti-inflammatory effect comes from the fact that inflammation activates the vagus nerves’ afferent fibers, which in turn signals the brain about the process, triggering an anti-inflammatory response called the inflammatory reflex [22].

The cholinergic anti-inflammatory pathway (CAP) is the main pathway activated in this response. It innervates the spleen through the efferent vagus nerve, and from the splenic nerve, it relays and acts on macrophages transforming adrenergic stimulation into a cholinergic signal, which exerts an anti-inflammatory effect [23]. Tracey [24] introduced the CAP concept and showed that the alpha7 subunit of the nicotinic acetylcholine receptor is required for inhibition of Tumor Necrosis Factor release from macrophages. The absence or failure of this mechanism results in excessive inflammatory responses, culminating in chronic conditions [22].

Important data also show that the increase in IL-6 in COVID-19 may be associated with poor prognosis, admission to the Intensive Care Unit, Acute Respiratory Distress Syndrome, respiratory failure, shock, multiple organ dysfunction, and risk of death, being a good marker to monitor these patients [25,26].

In a meta-analysis by Coomes and Haghbayan [26] it was reported that IL-6 levels were 2.9-fold higher in more critically ill patients when compared to milder cases. The same is reported for CRP, as it was observed that higher concentrations of this were associated with lower oxygen saturation, higher temperatures, higher platelet, and white blood cell counts, increased ferritin, and high levels of d-dimer. In addition to increased likelihood of having venous thromboembolism, acute kidney injury, severe illness, and mortality [27].

Similar to our results, Tracey [22] also did not observe the reduction of IL-10 by VNS. Non-inhibition of IL-10 is an advantage, as it is one of the most potent anti-inflammatory cytokines to help fight the COVID-19 cytokine storm [23,24].

Regarding cortisol, there was no difference after taVNS. Cortisol plays a relevant role in the body’s metabolic reaction to stress [28]. When its level is high, it can restrict immune responses to prevent excess inflammation [29,30].

### 4.2. Autonomic Modulation

We found no significant differences in HF, LF, and LF/HF ratio outcomes for both groups. However, satisfactory results of taVNS on autonomic control have already been observed, but in healthy individuals [11].

Monitoring vagal tone in patients with COVID-19 may be essential, as it can be used as a predictive marker of the course of COVID-19 disease. This is because patients with a very low vagal tone at the onset of symptoms may be at high risk of developing a dysregulated pro-inflammatory response during infection, leading to sudden death or transfer to the intensive care unit [11,30].

Annane et al. [31] reported that high concentrations of catecholamines and impaired sympathetic modulation, ordinary in patients with septic shock, suggesting that central autonomic regulatory impairment contributes to circulatory failure. Thus, it is possible that in patients with COVID-19 a dysfunction in autonomic tone for cytokine release syndrome and multiorgan damage are related [32].

It is worth considering that this was the first study documented to date that has evaluated the effect of taVNS on HRV in people hospitalized with COVID-19. This relationship had not been observed with the findings of inflammatory mediators.

### 4.3. Clinical Symptoms

We found no difference in clinical symptoms after taVNS in both groups. We emphasize that participants were bedridden, which may contribute to increased pain, fatigue, and muscle weakness. The proportion of deaths was similar between the groups. All participants who died had comorbidities and were intubated, with the cause of death reported as COVID-19 infection. For memory and attention, taVNS has already been effectively observed [33]. However, we did not observe differences between the groups, and some participants reported worsening of these variables after the fourth month of diagnosis.

However, Staats et al. [34] reported two cases of patients with COVID-19. In case 1, the patient used nVNS to expedite symptomatic recovery at home after hospital discharge and was able to discontinue the use of opioid medications and cough suppressants; in case 2, the patient experienced immediate and consistent relief from symptoms of chest tightness and shortness of breath, as well as an improved ability to clear the lungs. Tarn et al. [21] observed an improvement in fatigue and immune responses in Sjögren’s syndrome after nVNS and Lai et al. [35] reported relief of acute pain for migraine after cervical nVNS.

Although we did not observe major clinical changes between the treated and untreated groups, the literature shows that intense and chronic inflammation can lead to encephalopathy, encephalitis, acute disseminated encephalomyelitis, myelitis, meningitis, ischemic infarctions, or cerebral venous thrombosis [36]. It is noteworthy that the etiology of encephalopathy in COVID-19 is mainly linked to central and peripheral nervous system damage by a cytokine storm, blood clots, or direct damage to specific receptors [37,38].

Furthermore, it has now been well established that both the innate and adaptive immune systems become dysregulated in depressed patients and that controlling inflammation may be of therapeutic benefit [39]. Two meta-analyses showed reliably higher levels of inflammatory markers in depression, namely IL-1β, IL-6, C-reactive protein (CRP), and TNF-α [40,41].

Thus, based on the psychological impact of the COVID-19 pandemic on psychiatric patients, Guo et al. [42] speak of the importance of targeting the cholinergic anti-inflammatory pathway and modulating brain circuits using taVNS based on what the literature reports on the benefit of the technique to treat COVID-19 and its associated cytokine storm.

Therefore, controlling inflammation may provide an overall long-term therapeutic benefit. This corroborates our findings regarding depression assessed after the end of treatment. Patients in the treated group showed a significant decrease in symptoms of depression when compared to the group not treated with taVNS. Although vagus nerve stimulation has already been approved by the US Food and Drug Administration (FDA) for the treatment of depression [43], it has been applied for the adjunctive treatment of resistant depression [44,45,46]. This is the first study that has evaluated its effect for depression for hospitalized patients with COVID-19.

In addition to the disease itself being a cause of depression [47], this symptom is the most common mental illness in hospitalized individuals. Studies show that the prevalence of depressive syndrome in hospitalized patients ranges from 15% to 56%. Despite the significant prevalence, depression is generally underdiagnosed and undertreated [48,49,50].

Thus, Lai et al. [51] report the importance of evidence-based assessments and interventions targeting mental health disorders in COVID-19, as these are scarce and Guo et al. [42] suggest that taVNS can be used as an adjunctive therapy for depressive symptoms during the COVID-19 pandemic; they, however, have not tested the therapy for this population.

The use of taVNS in patients with COVID-19 should still be explored, due to the close relationship between the inflammatory process (a characteristic of COVID-19) and depression. There is evidence showing a sensitivity of the insula and striatum to changes in peripheral inflammation in depression in rodents and humans [52].

In a recent study, CRP level was negatively correlated with amygdala–ventromedial prefrontal cortex connectivity in depressed patients with high levels of inflammation and anxiety symptoms [53]. Nusslock et al. [54] found that higher levels of inflammatory biomarkers were associated with lower connectivities in both the emotional network and the core executive network in urban African American youth, suggesting that inflammation or neuroimmunology may be involved in the pathogenesis of emotional health problems and physical.

These studies show that systemic inflammation is associated with activity in the striatum with reward-related neural and interoceptive circuits and provide evidence of physiological subtypes within depression.

Therefore, depression in COVID-19 may have different causes than resistant depression, which is already treated with taVNS with obvious effects. Thus, there is a need for clinical evidence of the use of taVNS in depression in people with COVID-19.

## 5. Conclusions

Based on this study’s results, taVNS can reduce CRP, IL-6, and depression in patients with COVID-19 but does not interfere in cardiac modulation, COVID-19 clinical symptoms, anxiety, memory, and attention levels.

## Figures and Tables

**Table 1:** Demographic Data of Participants Diagnosed with COVID-19 (N = 52).

Variables	a-taVNS (N = 26)	s-taVNS (N = 26)
**Participants**		
Sex, male *n* (%)	16 (61%)	10 (38%)
Age (years), mean (SD)	53 (17)	57 (16)
Weight (kg), mean (SD)	83 (13)	86 (17)
Height (m), mean (SD)	1.67 (7)	1.66 (7)
BMI (Kg/m^2^), mean (SD)	30 (4)	31 (6)
SBP (mmHg), mean (SD)	124 (17)	126 (29)
DBP (mmHg), mean (SD)	78 (10)	75 (12)
HR (bpm), mean (SD)	83 (13)	80 (16)
IL-6 (pg/mL), mean (SD)	32 (28)	43 (38)
IL-10 (pg/mL), mean (SD)	10 (9)	8 (6)
CRP (mg/dL), mean (SD)	9 (7)	11 (10)
Cortisol (ug/dL), mean (SD)	21 (17)	25 (21)
LF (n.u), mean (SD)	52 (27)	50 (31)
HF (n.u), mean (SD)	48 (27)	50 (31)
HF/LF ratio, mean (SD)	3 (5)	3 (4)
Anxiety, mean (SD)	8 (3)	5 (3)
Depression, mean (SD)	5 (4)	3 (3)
Onset of Symptoms (days), mean (SD)	9 (2)	9 (2)
Death (n)	4	6
**Comorbidities**		
AH, *n* (%)	12 (46%)	15 (58%)
Diabetes mellitus, *n* (%)	7 (27%)	7 (27%)
Obesity, *n* (%)	11 (42%)	13 (50%)
COPD, *n* (%)	1 (4%)	7 (27%)
Smokers, *n* (%)	1 (4%)	1 (4%)
**Vaccinated**, *n* (%)	21 (81%)	15 (58%)
*Pfizer, n (%)*		
1st dose	2 (8%)	1 (4%)
2nd dose	1 (4%)	0 (0%)
*CoronaVAC, n (%)*		
1st dose	12 (46%)	10 (38%)
2nd dose	9 (35%)	10 (38%)
*AstraZeneca, n (%)*		
1st dose	6 (23%)	3 (12%)
2nd dose	4 (15%)	2 (8%)
*Janssen, n (%)*	1 (4%)	1 (4%)

Legend: Values presented in absolute frequency, mean, and standard deviation (SD). BMI (Body mass index), SBP (Systolic blood pressure), DPB (Diastolic blood pressure), HR (Heart Rate), mmHg (millimeter per mercury), IL-6 (Interleukin-6), IL-10 (Interleukin-10), CRP (C-reactive protein), LF (low frequency), HF (high frequency), LF/HF (low frequency and high frequency ratio), bpm (beat per minute), AH (arterial hypertension), COPD (chronic obstructive pulmonary disease).

**Table 2:** Results of Anxiety and Depression Levels. Comparison of Crude and Adjusted Treatment Effects for Unbalanced Variables at Baseline (Vaccination Status, Anxiety Score, Sex, and Body Mass Index).

a-taVNS (N = 26)					s-taVNS (N = 26)				
Variable	Baseline	Post	Raw Changes within the Group	Adjusted Changes within the Group #	Baseline	Post	Raw Changes within the Group	Adjusted Changes within the Group #	Adjusted Difference between Groups ##
Anxiety	8.00 (3.5)	5.3 (3.7)	−2.7 (4.3)	2.5 (4.6)	5.5 (3.4)	4.7 (4.6)	−1.0 (3.7)	−1.2 (4.6)	−1.27
									(−4.1 to 1.5)
Depression	4.6 (4.0)	4.0 (5.0) *	−1.0 (3.8)	−1.8 (4.2)	3.2 (3.0)	3.1 (3.8)	0.2 (3.6)	1.0 (4.2)	−2.85
									(−5.4 to −0.3)

Note: Data reported as mean (SD), statistical comparison using multiple linear regression. # Adjusted for unbalanced variables at baseline (vaccination status, anxiety score, sex, and body mass index) (Table 1). ##Adjusted for unbalanced variables at baseline and reported as mean difference (IC 95%). * *p* < 0.05.

**Table 3:** Results from Clinical Global Impression Scale (CGI) Memory and Attention.

	a-taVNS (N = 20)								s-taVNS (N = 20)								
Outcome	7 d	14 d	30 d	60 d	90 d	120 d	150 d	180 d	7 d	14 d	30 d	60 d	90 d	120 d	150 d	180 d	Difference between Groups SD (95% CI)
Memory	4.15 (0.33)	3.95 (0.33)	4 (0.33)	3.45 (0.33)	3.1 (0.33)	3.4 (0.33)	4.05 (0.33)	4.15 (0.33)	4.05 (0.33)	4.10 (0.33)	3.5 (0.33)	3.75 (0.33)	3.9 (0.33)	3.85 (0.33)	3.85 (0.33)	3.95 (0.33)	−0.90 (−0.42 to 0.24)
Attention	3.9 (0.34)	3.9 (0.34)	3.85 (0.34)	3.55 (0.34)	3.35 (0.34)	3.6 (0.34)	4.25 (0.34)	4.3 (0.34)	3.85 (0.34)	3.7 (0.34)	3.5 (0.34)	3.6 (0.34)	3.75 (0.34)	3.8 (0.34)	3.75 (0.34)	3.65 (0.34)	0.14 (−0.20 to 0.47)

Legend: Values expressed as mean and standard deviation (SD); the test used was the Generalized Estimating Equations (EEG) test. d (Days).

## Data Availability

Not applicable.

## Appendix A

Study flowchart. Transcutaneous auricular vagus nerve stimulation (taVNS); Interleukin 6 (IL-6); Interleukin 10 (IL-10); C-reactive protein (CRP); Heart rate variability (HRV); Hospital Anxiety and Depression Scale (HAD). * Analysis 1: 26 participants had their blood collected for the analysis of inflammatory markers and had their heart rate variability analyzed. For the assessment of HAD and clinical symptoms, 20 participants answered the questionnaire, since six of them were intubated in both groups.

## Appendix B. Medications Intake during Trial (*n* = 52)

**Medication**	**s-taVNS (*n* = 26**) **Number (%)**	**a-taVNS (*n* = 26)** **Number (%)**	***p*-Value**
Antibiotics	20 (76.9)	23 (88.5)	0.23
Nonsteroidal anti-inflammatory drugs (NSAIDs)	12 (46.2)	18 (69.2)	0.09
Corticosteroids	24 (92.3)	25 (96.2)	0.54
Anticoagulants	21 (80.8)	25 (96.2)	0.07
Antihypertensives	21 (80.8)	26 (100.0)	0.08
Bronchodilator	8 (30.8)	13 (50.0)	0.16
Triglyceride Lowering Drugs	3 (11.5)	6 (23.1)	0.30
Antihistaminic	1 (3.8)	8 (30.8)	0.06
Anesthetic drugs	22 (84.6)	26 (100.0)	0.06
Antithyroid drugs	1 (3.8)	3 (11.5)	0.29
Analgesic	12 (46.2)	17 (65.4)	0.16
Hypoglycemic drugs	10 (38.5)	16 (61.5)	0.09
Vasopressors	6 (23.1)	12 (46.2)	0.08
Gastrointestinal drugs *	10 (38.5)	26 (100.0)	0.001
Antipsychotics	4 (15.4)	6 (23.1)	0.46
Expectorants	0 (0.0)	2 (7.7)	0.17
Antidepressants	2 (7.7)	3 (11.5)	0.63
Antifungal drugs	1 (3.8)	5 (19.2)	0.09
Uric acid lowering agent	0 (0.0)	2 (7.7)	0.14
Antiepileptics	0 (0.0)	2 (7.7)	0.14
Antiseptics	0 (0.0)	1 (3.8)	0.30
* laxants, antiflatulent, or proton pump inhibitor.			
